# Management of intrathoracic and cervical anastomotic leakage after esophagectomy for esophageal cancer: a systematic review

**DOI:** 10.1186/s13017-019-0235-4

**Published:** 2019-04-04

**Authors:** Moniek H. P. Verstegen, Stefan A. W. Bouwense, Frans van Workum, Richard ten Broek, Peter D. Siersema, Maroeska Rovers, Camiel Rosman

**Affiliations:** 1 Department of Surgery, Radboudumc, Geert Grooteplein Zuid 10, 6525 GA Nijmegen, The Netherlands; 2 Gastroenterology and Hepatology, Radboudumc, Geert Grooteplein Zuid 10, 6525 GA Nijmegen, The Netherlands; 3 Operating Rooms and Health Evidence, Radboudumc, Geert Grooteplein Zuid 10, 6525 GA Nijmegen, The Netherlands

**Keywords:** Anastomotic, Leakage, Intrathoracic, Cervical, Esophagectomy, Treatment

## Abstract

**Background:**

Anastomotic leakage (0–30%) after esophagectomy is a severe complication and is associated with considerable morbidity and mortality. The aim of this study was to determine which treatment for anastomotic leakage after esophagectomy have the best clinical outcome, based on the currently available literature.

**Methods:**

A systematic literature search was performed in Medline, Embase, and Web of Science until April 2017. All studies reporting on the specific treatment of cervical or intrathoracic anastomotic leakage following esophagectomy with gastric tube reconstruction for esophageal or cardia cancer were included. The primary outcome parameter was postoperative mortality. Methodological quality was assessed by the Newcastle-Ottawa Quality Assessment Scale.

**Results:**

Nineteen retrospective cohort studies including 273 patients were identified. Methodological quality of all studies was poor to moderate. Mortality rates of intrathoracic anastomotic leakages in the treatment groups were as follows: conservative (14%), endoscopic stent (8%), endoscopic drainage (8%), endoscopic vacuum-assisted closure system (0%), and surgery treatment group (50%). Mortality rates of cervical anastomotic leakages in the treatment groups were as follows: conservative (8%), endoscopic stent (29%), and endoscopic dilatation (0%).

**Discussion:**

Due to small cohorts, heterogeneity between studies, and lack of data regarding leakage characteristics, no evidence supporting a specific treatment for anastomotic leakage after esophagectomy was found. A severity score based on leakage characteristics instead of treatment given is essential for determining the optimal treatment of anastomotic leakage. In the absence of robust evidence-based treatment guidelines, we suggest customized treatment depending on sequelae of the leak and clinical condition of the patient. PrDepartment of Surgery, Radboudumc, P.O.B. 9101/618 NLactical advices are provided.

**Trial registration:**

Registration number PROSPERO: CRD42016032374.

**Electronic supplementary material:**

The online version of this article (10.1186/s13017-019-0235-4) contains supplementary material, which is available to authorized users.

## Background

The incidence of esophageal carcinoma is increasing. Yearly, 450,000 patients are diagnosed with esophageal cancer worldwide, and approximately 135,000 (30%) of these patients will undergo curative resection [1, 2]. Anastomotic leakage (0–30%) is a severe complication after esophagectomy [3, 4]. The occurrence of anastomotic leakage is associated with a prolonged length of stay on the intensive care unit (ICU) and within the hospital, a reduced quality of life, high costs, and an increased mortality rate [4–7].

The severity of anastomotic leakage ranges from asymptomatic to full-blown sepsis with multiple organ failure. Factors that may influence the severity of the anastomotic leakage are the location of the anastomosis (intrathoracic or cervical), the size and circumference of the defect, and the extent of contamination [8]. Factors that influence the severity of anastomotic leakage might also impact the most appropriate treatment strategy. Treatment of anastomotic leakage ranges from “conservative” (nil by mouth, antibiotics, gastric drainage, enteral or parenteral feeding, and drainage through percutaneous tubes) to endoscopic treatment with stents or endoscopic vacuum-assisted closure (VAC) devices, and surgery [9]. However, no generally accepted treatment strategy for the treatment of anastomotic leakage after esophagectomy currently exists [10].

The aim of this study was to determine which treatment for a cervical or intrathoracic anastomotic leakage after esophagectomy with gastric tube reconstruction has the best clinical outcome, based on literature findings.

## Methods

This review was registered in the PROSPERO database for systematic reviews under number CRD42016032374 [11]. It was performed according to the Preferred Reporting Items for Systematic Reviews and Meta-analyses (PRISMA) guidelines, and the PRISMA checklist is shown online in Additional file 1: Appendix 1 [12]. A systematic literature search was performed in Medline, Embase, Web of Science, and the Cochrane Library for studies published from inception to April 24, 2017. The search terms used were *esophageal neoplasm* or *esophagectomy*, and *anastomotic leak* or *gastrointestinal leak*, and synonyms, and were restricted to title, abstract, and keywords (see Additional file 1: Appendix 2 for the full electronic search strategy). There were no restrictions regarding language, year of publication, or publication status.

### Study selection and data extraction

Titles, abstracts, and subsequently full-text articles were screened independently by two authors (M.V. and S.B.), and eligibility was assessed. All studies concerning the treatment of cervical and/or intrathoracic anastomotic leakage after esophagectomy for cancer of the esophagus or gastric cardia with gastric tube reconstruction were included. Studies not reporting the location of the anastomosis were excluded. Studies primarily investigating the treatment of other disorders affecting interruption of esophageal integrity such as iatrogenic injuries, spontaneous ruptures, conduit line dehiscence, or necrosis of the gastric conduit were excluded. In addition, review articles, editorials, case reports or cohort studies including fewer than five patients per specific treatment strategy, animal studies, and studies in children were excluded. Disagreement on eligibility was resolved after discussion. Reference lists of all included articles were screened manually to identify initially missed, but relevant studies. Data was extracted by M.V. and S.B. independently and entered into an electronic database (IBM SPSS for Windows version 22.0, Armonk, NY).

### Assessment of methodological quality

The risk of bias was assessed using the Newcastle-Ottawa Quality Assessment Scale for cohort studies [13]. This scale rates studies on 3 sources of bias (selection, comparability, and outcome) based on 8 criteria. Each criterion is awarded with 1 star except comparability, which is awarded a maximum of 2 stars. For this systematic review, studies scoring 7–9 stars were considered to be of high methodological quality, studies scoring 4–6 stars were considered to be of moderate methodological quality, and studies scoring 1–3 stars were considered to be of poor methodological quality. The methodological quality of all included studies was assessed independently by 2 authors (M.V. and S.B.). Disagreements were resolved by discussion and consensus with a third reviewer (C.R.).

### Study characteristics, leakage characteristics, and outcome parameters

The following study characteristics were extracted: first author, year of publication, country of origin, number of included patients, study design (prospective or retrospective), type of modality used to diagnose the leakage, type of operation, and location of the anastomosis.

Data regarding leak characterization included the following: time from surgery to diagnosis of the leakage, time from diagnosis to treatment of the leakage, the mean interval of the leakage treatment, circumference of the leakage (0–25%, 25–50%, 50–75%, and 75–100%), length of the leakage (in centimeters), gastric conduit overall condition (vital, ischemic, or necrotic), and extent of the contamination (i.e., none, mediastinal fluid collections, or pleural fluid collections).

The primary outcome parameter was mortality rate. The secondary outcome parameters were as follows: success rate (when not defined by the author, defined as alive and no persisting leakage during time of follow-up), severe complications occurring after anastomotic leakage treatment (Clavien-Dindo [14] ≥ 3), reintervention rate (all surgical, endoscopic, and radiological reinterventions), reoperation rate, new onset of (multiple) organ failure, hospital length of stay, ICU length of stay, and quality of life.

### Analysis

Due to heterogeneity between studies, no meta-analysis could be performed. The weighted percentages and means were calculated to summarize the treatment outcomes for each subgroup.

## Results

### Included studies

**Fig. 1:**
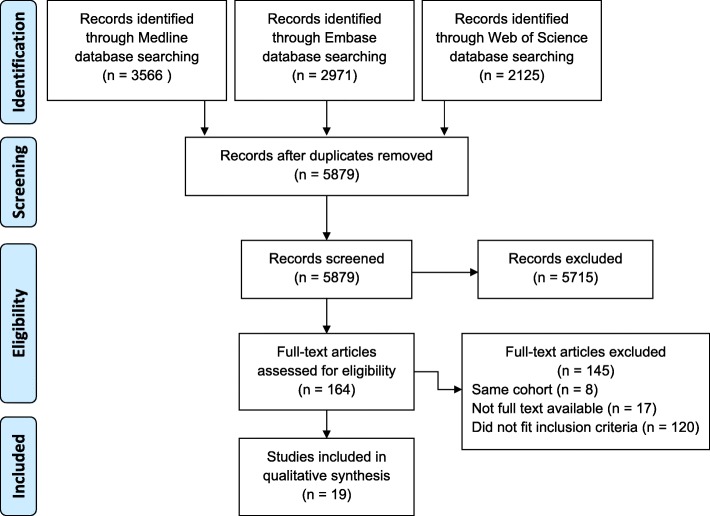
Summary of screening and selection process, PRISMA diagram

Nineteen studies, including a total of 273 patients, met the inclusion criteria of this systematic review. A summary of the screening and selection process is shown in Fig. 1.

### Study characteristics

All 19 included studies were retrospective cohort studies [15–33], of which 2 studies were comparative cohort studies (Table 1) [15, 16]. An intrathoracic anastomosis was performed in 200 patients and a cervical anastomosis in 73 patients. The incidence of anastomotic leakage could be calculated in only 2 studies; the incidence rates were 1% and 17%, respectively [16, 20]. The average age of the patients was described in 6 studies; the weighted mean was 58.0 years (range 28–92 years) [16, 20, 21, 28, 31, 33]. Neo-adjuvant treatment was reported in 1 study, in which none was given [24]. Tumor characteristics were reported in 2 studies; in 1 study, stage I esophageal cancer was found in 3 patients, stage IIA in 9, stage IIB patients in 11 patients, and stage III in 5 patients [33]. In 1 study, 8 patients were diagnosed with adenocarcinoma and 2 patients with squamous cell carcinoma [31]. In 3 studies, operations were performed minimally invasively [15, 25, 30].

**Table 1:** General study characteristics

Author	Country	Design	Patients (*n*)	Type of operation	Outcomes reported
Intrathoracic					
Griffin 2001 [18]	UK	NC	13	NA	Mortality/success/complication/reoperation
Holscher 2003 [19]	DE	NC	6	Ivor Lewis	Mortality/success/complication/reoperation
Hunerbein 2004 [21]	DE	NC	9	Transhiatal or Ivor Lewis	Mortality/success/complication/reintervention/reoperation/hospital and ICU stay
Kauer 2008 [23]	DE	NC	10	Ivor Lewis	Mortality/success/complication/reintervention/reoperation
Tuebergen 2008 [31]	DE	NC	18	Ivor Lewis	Mortality/success/reintervention/reoperation/hospital stay
Qin 2010 [29]	CN	NC	5	Left thoracotomy	Mortality/success/hospital stay
Hu 2011 [20]	CN	C	23	Ivor Lewis	Mortality/success/reoperation/hospital stay/ICU stay
Hu 2011 [20]	CN	C	17	Ivor Lewis	Mortality/success/reoperation/hospital stay/ICU stay
Jiang 2011 [22]	CN	C	7	Left thoracotomy	Mortality/success/complication/hospital stay
Jiang 2011 [22]	CN	C	25	Left thoracotomy	Mortality/success/complication/hospital stay
Yin 2012 [33]	CN	NC	28	Ivor Lewis	Mortality/success/complication/reintervention
Al-Issa 2014 [15]	DK	NC	15	Ivor Lewis (some MIE)	Mortality/success/complication/reintervention/hospital stay
Bludau 2014 [17]	DE	NC	5	Ivor Lewis	Mortality/success
Shuto 2017 [30]	JP	NC	19	Ivor Lewis (some MIE)	Mortality/success/complication/reintervention
Cervical					
Orringer 1986 [28]	US	NC	5	Orringer	Mortality/success/complication/reintervention
Bhasin 2000 [16]	IN	NC	8	Orringer	Mortality/success/complication/reintervention
Korst 2005 [24]	US	NC	13	McKeown or left thoracotomy	Mortality/success/complication/reintervention/hospital stay
Lindeman 2008 [27]	AT	NC	6	McKeown or Orringer	Mortality/success/complication/reintervention
Larburu 2013 [25]	ES	NC	9	McKeown or Orringer (both MIE)	Mortality/success/complication/reoperation
Leenders 2013 [26]	NL	NC	9	Orringer	Mortality/success/complication/reintervention/reoperation/hospital stay
Van Rossum 2017 [32]	NL	NC	23	McKeown	Mortality/success/complication/reintervention/reoperation/hospital and ICU stay

Complication = severe complication rate, hospital stay = hospital length of stay, ICU stay = ICU length of stay, Ivor Lewis = esophagectomy by laparotomy and thoracotomy with intrathoracic anastomosis, McKeown = esophagectomy by laparotomy and thoracotomy with cervical anastomosis, MIE = minimally invasive esophagectomy, mortality = mortality rate, Orringer = transhiatal esophagectomy with cervical anastomosis, reintervention = reintervention rate, reoperation = reoperation rate, success = success rate

*C* comparative, *NA* not available, *NC* non-comparative

*AT* Austria, *CN* China, *DE* Germany, *DK* Denmark, *ES* Spain, *IN* India, *JP* Japan, *NL* Netherlands, *UK* United Kingdom, *US* United States of America

In 17 studies, the following modalities were used to diagnose anastomotic leakage: contrast swallow examination (*n* = 15 studies), endoscopy (*n* = 11 studies), and computed tomography (CT) scan (*n* = 10 studies). In 6 studies, a conservative treatment was performed consisting of the administration of antibiotics, nil by mouth, enteral tube feeding, gastric drainage and (percutaneous) drainage of the mediastinum, thoracic cavity, and/or wound [18, 20, 22, 24, 29, 32]. Patients were treated by endoscopic vacuum-assisted wound closure (VAC) system in 1 study [17], by endoscopic drainage in 4 studies [20, 22, 30, 33], and by endoscopic stent placement in 7 studies [15, 21, 23, 25–27, 31]. In 2 studies (*n* = 13 patients), endoscopic balloon dilatation was performed to improve the healing of the leak [16, 18]. One study performed a re-thoracotomy with revision of the anastomosis (Table 2) [19]. Quality of life was not reported in any of the studies. Severe complications were described in 15 studies [15, 16, 18, 19, 21–28, 30, 32, 33], but none of the included studies reported the severity of complications according to the Clavien-Dindo scale.

**Table 2:** Clinical study characteristics

Author	Patients (*n*)	Diagnosis of leakage	General treatment	Investigational treatment
Intrathoracic				
Griffin 2001 [18]	13	Contrast/endoscopy	Antibiotics, nil by mouth, enteral feeding tube, gastric, mediastinal, and thoracic drainage	Conservative treatment
Holscher 2003 [19]	6	Contrast/endoscopy/CT	Gastric drainage	Surgery
Hunerbein 2004 [21]	9	Contrast/endoscopy	Antibiotics, thoracic drainage	Stent (plastic)
Kauer 2008 [23]	10	Contrast/endoscopy	Mediastinal drainage	Stent (SEMS)
Tuebergen 2008 [31]	18	Contrast/endoscopy	Antibiotics, nil by mouth, enteral feeding tube, gastric, mediastinal, and thoracic drainage	Stent (SEMS)
Qin 2010 [29]	5	Contrast	Antibiotics, nil by mouth, enteral feeding tube, gastric, mediastinal, and thoracic drainage	Conservative treatment
Hu 2011 [20]	23	Contrast/CT	Antibiotics, nil by mouth, enteral feeding tube, thoracic drainage	Endoscopic drainage
Hu 2011 [20]	17	Contrast/CT	Antibiotics, nil by mouth, enteral feeding tube, gastric and thoracic drainage	Conservative treatment
Jiang 2011 [22]	7	Contrast	Nil by mouth, enteral feeding tube, gastric and thoracic drainage	Conservative treatment
Jiang 2011 [22]	25	Contrast	Nil by mouth, enteral feeding tube, thoracic drainage	Endoscopic drainage
Yin 2012 [33]	28	Contrast/CT	Nil by mouth, enteral feeding tube, gastric drainage	Endoscopic drainage
Al-Issa 2014 [15]	15	Endoscopy/CT	Antibiotics, nil by mouth, enteral feeding tube, thoracic drainage	Stent (SEMS)
Bludau 2014 [17]	5	Contrast/endoscopy/CT	NA	Endoscopic VAC
Shuto 2017 [30]	19	Contrast/endoscopy/CT	Antibiotics, nil by mouth, enteral feeding tube, gastric, mediastinal, and thoracic drainage	Endoscopic drainage
Cervical				
Orringer 1986 [28]	5	NA	Nil by mouth, enteral feeding tube, gastric drainage, wound drainage	Endoscopic dilatation
Bhasin 2000 [16]	8	Contrast	Nil by mouth, enteral feeding tube, wound drainage	Endoscopic dilatation
Korst 2005 [24]	13	Contrast/endoscopy/CT	Antibiotics, wound drainage	Conservative treatment
Lindeman 2008 [27]	6	NA	Nil by mouth, wound drainage	Stent (SEMS)
Larburu 2013 [25]	9	Contrast/endoscopy/CT	NA	Stent (SEMS)
Leenders 2013 [26]	9	NA	Wound drainage or percutaneous drainage	Stent (SEMS)
Van Rossum 2017 [32]	23	Contrast/endoscopy/CT	Nil by mouth, enteral feeding tube, gastric drainage, wound drainage	Conservative treatment

Conservative treatment = antibiotics/nil by mouth/enteral feeding tube/gastric drainage/mediastinal drainage/thoracic drainage/percutaneous drainage/wound drainage; contrast = contrast swallow examination

*CT* computerized tomography, *NA* not available, *SEMS* self-expandable metallic stent

### Methodological quality

Methodological quality of the included studies is described in Table 3. The quality of the included studies was poor in 9 studies [15, 18, 19, 23, 24, 26–29] and moderate in 10 studies [16, 17, 20–22, 25, 30–33]. Ten of the 19 studies reported on selected cases (i.e., excluding the critically ill and intensive care patients) [15, 16, 18, 26–31, 33], which is not representative of the general hospitalized population with an anastomotic leakage after esophageal resection.

**Table 3:** Methodological quality

Author	Selection Representativeness (maximum: one star)	Selection Selection (maximum: one star)	Selection Ascertainment (maximum: one star)	Selection Outcome of interest (maximum: one star)	Comparability Comparability (maximum: two stars)	Outcome Assessment of outcome (maximum: one star)	Outcome Duration of follow-up (maximum: one star)	Outcome Adequacy of follow-up (maximum: one star)	Total Score (maximum: 9 stars)
Intrathoracic									
Griffin 2001 [18]	0	NA	0	*	NA	0	NA	NA	1
Holscher 2003 [19]	*	NA	*	*	NA	0	NA	NA	3
Hunerbein 2004 [21]	*	NA	*	*	NA	0	*	*	5
Kauer 2008 [23]	*	NA	0	*	NA	*	NA	NA	3
Tuebergen 2008 [31]	0	NA	*	*	NA	*	*	*	5
Qin 2010 [29]	0	NA	*	*	NA	*	NA	NA	3
Hu 2011 [20]	*	NA	*	*	**	0	NA	NA	5
Jiang 2011 [22]	*	NA	*	*	*	0	*	*	6
Yin 2012 [33]	0	NA	*	*	NA	0	*	*	4
Al-Issa 2014 [15]	0	NA	*	*	NA	*	NA	NA	3
Bludau 2014 [17]	*	NA	0	*	NA	*	0	*	4
Shuto 2017 [30]	0	NA	*	*	NA	*	0	*	4
Cervical									
Orringer 1986 [28]	0	NA	0	*	NA	0	*	*	3
Bhasin 2000 [16]	0	NA	*	*	NA	*	0	*	4
Korst 2005 [24]	*	NA	0	*	NA	*	NA	NA	3
Lindeman 2008 [27]	0	NA	*	*	NA	0	0	0	2
Larburu 2013 [25]	*	NA	*	*	NA	0	NA	*	4
Leenders 2013 [26]	0	NA	0	*	NA	0	NA	NA	1
Van Rossum 2017 [32]	*	NA	*	*	NA	0	*	*	5

* = one star, ** = two stars

*NA* not available

### Characteristics of anastomotic leakage

The mean time from surgery to diagnosis of the anastomotic leakage was reported in 9 studies and was 9 days (range 2–30) [16, 19–22, 27–29, 33]. Only 2 studies reported the time from diagnosing the leakage and the treatment of it (mean 8 days (range 0–20)) [16, 26]. The mean duration of the leakage treatment was reported in 11 studies and was 34 days [16, 17, 21, 22, 24, 26–29, 31, 33]. Two studies reported the percentage of the circumference of the leak; 1 study included patients with a defect less than 2/3 of the circumference [31], the other study with a defect between 10 and 30% of the circumference [27]. The length of the leak in centimeters was reported in 2 studies, 1 study only included patients with a leak > 1 cm [15] and 1 study only included patients with a leak > 0.5 cm [23]. No studies reported data on the general condition of the gastric tube. Five studies reported data on contamination that was caused by the leak but used different descriptions (e.g., small vs. extended, cervical vs. intrathoracic manifestation of the cervical leakages) [21, 22, 24, 25, 32]. In 4 patients, the leakage was associated with fistula formation to the airways (*n* = 2) or gastric conduit necrosis (*n* = 2) [25, 31]. None of the studies reported outcomes per anastomotic leakage characteristic, and therefore, no further analysis of the effectiveness of different treatment modalities per leakage characteristic could be performed.

### Outcomes of anastomotic leakage treatment

#### All studies

The overall mortality was 11% (31/273 patients). The mortality rates were as follows: 12% (9/78 patients) in the conservative group, 14% (11/76 patients) in the endoscopic stent group, 8% (8/95 patients) in the endoscopic drainage group, 0% (0/5 patients) in the endoscopic VAC therapy, 0% (0/13 patients) in the endoscopic dilatation group, and 50% (3/6 patients) in the surgical treatment group (Table 4). Only two studies reported the new onset of (multiple) organ failure, respectively 14% (1/7 patients) and 50% (3/6 patients) of patients [19, 22]. Other outcome parameters are reported in Table 5.

**Table 4:** Primary outcome: mortality rate

	Studies, *n*	Included patients, *n*	Mortality, *n* (%)
Overall			
Conservative	6	78	9 (12%)
Endoscopic			
Stent	7	76	11 (14%)
Drainage	4	95	8 (8%)
Endoscopic VAC	1	5	0 (0%)
Dilatation	2	13	0 (0%)
Surgical	1	6	3 (50%)
Intrathoracic anastomotic leakage			
Conservative	4	42	6 (14%)
Endoscopic			
Stent	4	52	4 (8%)
Drainage	4	95	8 (8%)
Endoscopic VAC	1	5	0 (0%)
Surgical	1	6	3 (50%)
Cervical anastomotic leakage			
Conservative	2	36	3 (8%)
Endoscopic			
Stent	3	24	7 (29%)
Dilatation	2	13	0 (0%)

*VAC* vacuum-assisted closure

**Table 5:** Other outcome parameters

	Studies, *n*	Included patients, *n*	Outcome, *n* (%)
Mortality rate			
Conservative	6	78	9 (12%)
Endoscopic			
Stent	7	76	11 (14%)
Drainage	4	95	8 (8%)
Endoscopic VAC	1	5	0 (0%)
Dilatation	2	13	0 (0%)
Surgical	1	6	3 (50%)
Success rate			
Conservative	6	78	69 (88%)
Endoscopic			
Stent	7	76	57 (75%)
Drainage	4	95	86 (91%)
Endoscopic VAC	1	5	5 (100%)
Dilatation	2	13	13 (100%)
Surgical	1	6	3 (50%)
Severe complications			
Conservative	4	56	9 (16%)
Endoscopic			
Stent	6	58	27 (47%)
Drainage	3	72	13 (18%)
Endoscopic VAC		NA	NA
Dilatation	2	13	5 (38%)
Surgical	1	6	3 (50%)
Reinterventions			
Conservative	2	36	5 (36%)
Endoscopic			
Stent	6	67	27 (40%)
Drainage	2	47	10 (21%)
Endoscopic VAC	NA	NA	NA
Dilatation	2	13	5 (38%)
Surgical	NA	NA	NA
Mean number of reinterventions			
Conservative	2	36	3
Endoscopic			
Stent	6	67	1
Drainage	2	47	1
Endoscopic VAC	NA	NA	NA
Dilatation	2	13	4
Surgical	NA	NA	NA
Reoperations			
Conservative	3	53	2 (4%)
Endoscopic			
Stent	4	46	6 (13%)
Drainage	1	23	1 (4%)
Endoscopic VAC	NA	NA	NA
Dilatation	NA	NA	NA
Surgical	1	6	2 (33%)
Hospital length of stay			
Conservative	4	65	41 days
Endoscopic			
Stent	4	51	37 days
Drainage	2	48	42 days
Endoscopic VAC	NA	NA	NA
Dilatation	NA	NA	NA
Surgical	NA	NA	NA
ICU length of stay			
Conservative	2	40	16 days
Endoscopic			
Stent	1	9	25 days
Drainage	1	23	12 days
Endoscopic VAC	NA	NA	NA
Dilatation	NA	NA	NA
Surgical	NA	NA	NA

*VAC* vacuum-assisted closure, *NA* not available

#### Subgroup—intrathoracic anastomotic leakage

The overall mortality after intrathoracic anastomotic leakage was 11% (21/200 patients). The mortality rates were as follows: 14% (6/42 patients) in the conservative group, 8% (4/52 patients) in the endoscopic stent group, 8% (8/95 patients) in the endoscopic drainage group, 0% (0/5 patients) in the endoscopic VAC therapy group, and 50% (3/6 patients) in the surgical treatment group (Table 4). The reintervention rate was higher in the endoscopic stent group compared to other treatment groups: 19 patients (37%) needed at least 1 reintervention, most often because of stent migration. Table 6 provides an overview of the other outcome measures.

**Table 6:** Outcomes intrathoracic anastomosis

	Studies (*n*)	Included patients (*n*)	Outcome
Mortality rate			
Conservative	4	42	6 (14%)
Endoscopic			
Stent	4	52	4 (8%)
Drainage	4	95	8 (8%)
Endoscopic VAC	1	5	0 (0%)
Surgical	1	6	3 (50%)
Success rate			
Conservative	4	42	36 (86%)
Endoscopic			
Stent	4	52	40 (77%)
Drainage	4	95	86 (91%)
Endoscopic VAC	1	5	5 (100%)
Surgical	1	6	3 (50%)
Severe complications			
Conservative	2	20	3 (15%)
Endoscopic			
Stent	3	34	14 (41%)
Drainage	3	72	13 (18%)
Endoscopic VAC	NA	NA	NA
Surgical	1	6	3 (50%)
Reinterventions			
Conservative	NA	NA	NA
Endoscopic			
Stent	4	52	19 (37%)
Drainage	2	47	10 (21%)
Endoscopic VAC	NA	NA	NA
Surgical	NA	NA	NA
Mean number of reinterventions			
Conservative	NA	NA	NA
Endoscopic			
Stent	4	52	1
Drainage	2	47	1
Endoscopic VAC	NA	NA	NA
Surgical	NA	NA	NA
Reoperations			
Conservative	2	30	1 (33%)
Endoscopic			
Stent	3	37	2 (5%)
Drainage	1	23	1 (4%)
Endoscopic VAC	NA	NA	NA
Surgical	NA	NA	2 (33%)
Hospital length of stay			
Conservative	3	29	64 days
Endoscopic			
Stent	3	42	38 days
Drainage	2	48	42 days
Endoscopic VAC	NA	NA	NA
Surgical			NA
ICU length of stay			
Conservative	1	17	34 days
Endoscopic			
Stent	1	9	25 days
Drainage	1	23	12 days
Endoscopic VAC	NA	NA	NA
Surgical	NA	NA	NA

*NA* not available

#### Subgroup—cervical anastomotic leakage

The overall mortality after cervical anastomotic leakage was 14% (10/73 patients). The mortality rates were as follows: 8% (3/36 patients) in the conservative treatment group, 29% (7/24 patients) in the endoscopic stent group, and 0% (0/13 patients) in the endoscopic dilatation group (Table 4). The reintervention and reoperation rates were 53% (8/15 patients) and 44% (4/9 patients) in the endoscopic stent group. Table 7 provides an overview of the other outcome measures.

**Table 7:** Outcomes cervical anastomosis

	Studies (*n*)	Included patients (*n*)	Outcome
Mortality rate			
Conservative	2	36	3 (8%)
Endoscopic			
Stent	3	24	7 (29%)
Dilatation	2	13	0 (0%)
Success rate			
Conservative	2	36	33 (92%)
Endoscopic			
Stent	3	24	17 (71%)
Dilatation	2	13	13 (100%)
Severe complications			
Conservative	2	36	6 (17%)
Endoscopic			
Stent	3	24	13 (54%)
Dilatation	2	13	5 (38%)
Reinterventions			
Conservative	2	36	5 (14%)
Endoscopic			
Stent	2	15	8 (53%)
Dilatation	2	13	5 (38%)
Mean number of reinterventions			
Conservative	2	36	3
Endoscopic			
Stent	2	15	1
Dilatation	2	13	4
Reoperations			
Conservative	1	23	1 (4%)
Endoscopic			
Stent	1	9	4 (44%)
Dilatation	NA	NA	NA
Hospital length of stay			
Conservative	2	36	22 days
Endoscopic			
Stent	1	9	36 days
Dilatation	NA	NA	NA
ICU length of stay			
Conservative	1	23	2 days
Endoscopic			
Stent	NA	NA	NA
Dilatation	NA	NA	NA

*NA* not available

## Discussion

This is the first systematic review summarizing the results of different treatment strategies for anastomotic leakage in patients after esophagectomy with gastric tube reconstruction.

Results on conservative, endoscopic, and surgical treatment were reported in 6 studies, 14 studies, and 1 study, respectively. The mean overall mortality rate was 11%. In studies reporting the outcome of conservative treatment, the mean mortality was 12%, in the stent placement group 14%, and in the endoscopic drainage group 8%. For endoscopic VAC, endoscopic dilatation, and surgical treatment, the mortality rate could not reliably be estimated due to a low number of patients reported.

### Strengths and limitations

Furthermore, this review reports factors that (may) influence the severity and outcome of an anastomotic leakage. A potential limitation of this review is that many studies were excluded based on title and abstract due to not reporting treatment results for cervical and intrathoracic leakage separately. Aware of the fact that more data on anastomotic leakage is available, we did not include these studies because it would not contribute in finding an answer to our research question. In addition, the methodological quality of the included studies was limited. Half of the studies reported results of a highly selected group of patients, which makes the external validity of these data weak. All studies were retrospective and included a limited number of patients. The objective of the present study was to investigate the treatment of anastomotic leakages; however, due to a lack of reported baseline characteristics and definitions of anastomotic leakages, it remains unclear whether these cohorts and leakage rates are comparable. Furthermore, additional treatments (e.g., nil by mouth, nutritional support, gastric drainage) which patients received alongside the investigational treatment were different between studies or not specified. Characterization of the leak and definitions of outcome parameters used were frequently lacking and not comparable between studies. Because of the heterogeneity of the included studies, performing a meta-analysis was deemed not scientifically and clinically relevant. Finally, multiple forms of bias were found in the data, i.e., in 13 studies, the follow-up length was not reported or too short to find long-term complications, i.e., stricture and fistula formation, or stent migration and 10 studies reported on selected cases (i.e., excluding the critically ill and intensive care patients).

### Clinical implications

Based on the currently available evidence, it is not possible to provide a uniform strategy for the treatment of anastomotic leakage after esophagectomy. Although achieving the aim of this systematic review was not entirely possible with the currently available evidence, this review is highly instrumental in exposing the limitations of the current evidence and therefore uncovering areas for future research. Firstly, it is important to separately report outcomes of intrathoracic and cervical anastomotic leaks, because evidence suggests that these are separate entities and probably necessitate different treatment strategies [4, 34]. Secondly, a uniform definition of anastomotic leakage, including factors that may influence the severity of anastomotic leakages and outcome parameters, should be described to make data transparent and comparable between studies. These factors may include length of the leak, circumference of the leak, condition of the gastric tube (vital, ischemic, necrotic), and contamination caused by the leak [35]. Adequate description of anastomotic leakage makes it possible to evaluate whether these factors actually contribute to leak severity and compare different treatment strategies. This may lead to an anastomotic leakage severity score. A score based on leakage characteristics, rather than a scoring system based on leakage therapy (e.g., the ECCG grading system [36]), is essential for providing clinicians an optimal treatment strategy for patients with an anastomotic leak. No evidence-based recommendations could be provided from the literature. For the current practice, we recommend, based on our experiences, all patients with an anastomotic leakage should be treated with intravenous antibiotics, nasogastric tube drainage, and where possible enteral feeding through a jejunal feeding tube or jejunostomy. In case of a cervical anastomosis, the neck wound should be opened. Additional interventions depend on the sequelae of the leak and the condition of the patient. Undrained collections of the mediastinum and thoracic cavity should be drained by surgical or radiological placed percutaneous drains and/or an endoscopic suction tube through the anastomotic defect. If drainage is insufficient or in case of more extensive contamination, a more aggressive strategy may be appropriate and drainage can be performed by video-assisted thoracoscopic surgery (VATS) or thoracotomy. In addition to drainage of fluid collections, the defect can be closed surgically or covered/closed with an endoscopically placed stent or E-VAC system. However, more data is needed to evaluate the effectiveness of these recommendations. In this review, we showed that there are multiple treatments and strategies to treat anastomotic leakage. Together with the incidence of anastomotic leakage, it is unlikely that single-center cohort studies will include enough patients to provide robust data for an anastomotic leakage treatment strategy. More detailed data from a larger cohort is urgently needed to provide an evidence-based treatment strategy for anastomotic leakage after esophagectomy. Currently, the TENTACLE study (TreatmENT of AnastomotiC Leakage after Esophagectomy), an international retrospective cohort study on patients with an anastomotic leakage after esophagectomy for esophageal cancer, is being performed (NCT03829098) [37]. This study includes standardized characteristics of an anastomotic leakage and has a standardized outcome. This study could provide answers to current issues as which factors determine the severity of the leakage and which treatment options have the best outcomes.

## Conclusions

Due to small cohorts in the included studies, heterogeneity between studies and lack of data regarding leakage characteristics, no evidence supporting a specific treatment for anastomotic leakage after esophagectomy was found. A severity score based on leakage characteristics instead of treatment given is needed for determining the optimal treatment of anastomotic leakage. In the absence of robust evidence-based treatment guidelines, we recommend an individualized treatment depending on sequelae of the leak and condition of the patient.

## Additional file

Additional file 1: **Appendix 1.** PRISMA 2009 checklist.** Appendix 2.** The electronic sea. (DOCX 108 kb)

## Abbreviations

CT: Computer tomography
ICU: Intensive care unit
MIE: Minimally invasive esophagectomy
NA: Not available
PRISMA: Preferred Reporting Items for Systematic Reviews and Meta-analyses
SEMS: Self-expandable metallic stent
VAC: Vacuum-assisted closure
